# The Oncogenic Role of microRNA-130a/301a/454 in Human Colorectal Cancer via Targeting Smad4 Expression

**DOI:** 10.1371/journal.pone.0055532

**Published:** 2013-02-05

**Authors:** Lin Liu, Jing Nie, Lin Chen, Guanglong Dong, Xiaohui Du, Xin Wu, Yun Tang, Weidong Han

**Affiliations:** 1 Department of General Surgery, PLA General Hospital, Beijing, China; 2 Department of Molecular Biology, PLA General Hospital, Beijing, China; Vanderbilt University Medical Center, United States of America

## Abstract

Transforming growth factor (TGF)-β/Smad signaling plays an important role in colon cancer development, progression and metastasis. In this study we demonstrated that the microRNA-130a/301a/454 family is up-regulated in colon cancer tissues compared to paired adjacent normal mucosa, which share the same 3′-untranslational region (3′-UTR) binding seed sequence and are predicated to target Smad4. In colorectal cancer HCT116 and SW480 cells, overexpression of miRNA-130a/301a/454 mimics enhances cell proliferation and migration, while inhibitors of these miRNAs affect cell survival. The biological function of miRNA-130a/301a/454 on colon cancer cells is likely mediated by suppression of Smad4, and the up-regulation of the miRNAs is correlated with Smad4 down-regulation in human colon cancers. Collectively, these results suggest that miRNA-130a/301a/454 are novel oncogenic miRNAs contributing to colon tumorigenesis by regulating TGF-β/Smad signaling, which may have potential application in cancer therapy.

## Introduction

Colon cancer is one of the most frequent cancers and a common cause of cancer-related deaths [1]. Due to the poor prognosis and distant invasion and migration, the overall incidence of colon cancer is approximately 5% and the 5-year survival rate of colon cancer patients is very low [2]. Thus, the identification of new targets for the development of non-conventional treatments is urgent and will take advantage of progress in the broad and deep understanding of the molecular pathogenesis of colon cancer.

MicroRNAs (miRNAs) are an extensive class of small noncoding RNAs (18–25 nt), with a significant impact on a number of biological processes, including development, differentiation, growth, metabolism, and tumorigenesis, through direct binding to the 3′ untranslational region (3′-UTR) of target mRNAs [3]–[5]. MicroRNAs can regulate gene expression by two modes, depending on the degree of complementarity with the mRNA targets, to suppress translation or induce mRNA degradation [6], [7]. MicroRNAs can function as tumor suppressors or oncogenes based on whether or not the miRNAs specifically target oncogenes or tumor suppressor genes [8]. Both oncogenic miRNAs and tumor suppressive miRNAs have been demonstrated and described in colon carcinogenesis and progression, such as upregulated miR-135, miR-21, miR-17-92, and miR-196a, and downregulated miR-34, miR-195, and miR-365 [9]–[13]. The expression profile of miRNAs is highly tissue and cell type specific, thus demonstrating the biological functional significance of an expressed miRNA [14]. However, elucidating the features of expression and roles of miRNAs in cancer biology, especially colon cancer, remains an ongoing process.

The microRNA-130ac/301ab/454/721 family has the same 3′-UTR binding seed sequence. Recently, miR-130a/301ab has been reported to be upregulated in several types of cancer, such as hepatocellular carcinoma, nonsmall cell lung cancer, chronic myeloid leukaemia, pancreatic cancer, and breast cancer [15]–[21]; however, miR-130a/301a is down-regulated in chronic lymphocytic leukemia and sickle cell anemia [22], [23], indicating the complexity and diversity of the roles of miR-130a/301a in tumorigenesis. Nevertheless, the pattern of expression and role of miR-130a/301a/454 in colon carcinogenesis remains unknown.

In this study we investigated the expression and roles of miR-130a/301a/454 in colon cancer development. We showed that miR-130a/301a/454 is up-regulated in clinically-resected human colon cancer tissues and colon cancer cell lines, and these miRNAs exhibit oncogenic properties in colon cancer cells *in vitro* and *in vivo*. The mechanisms underlying the roles of miR-130a/301a/454 in colon cancer development were also investigated. Thus, our data highlight the significance of the miR-130a/301a/454/family in cancer pathogenesis and suggest a potential application in cancer therapy.

## Materials and Methods

### Patients and tissue samples

Surgically-removed colon cancer tissues and paired adjacent normal mucosa tissues were collected from 35 colon cancer patients at the General Hospital of PLA (Beijing, China), and were used for quantitative reverse transcription-polymerase chain reaction (qRT-PCR) and immunoblotting analysis. The surgically-resected tissues were quickly frozen in liquid nitrogen until analysis. All samples were obtained with the informed consent of the patients and the experiments were approved by the Ethics Committee of the General Hospital of PLA. All participants provided written informed consent to participate in this study.

### RNA extraction and qRT-PCR

Total RNA, including miRNA, was isolated using the TRIzol reagent (Invitrogen) according to the manufacturer's instruction. For miRNA analysis, poly-A real-time qRT-PCR was used. The isolated RNA was polyadenylated using poly-A polymerase (New England Biolabs) according to the manufacturer's protocol. Then, the poly-A tailed RNA was reverse transcribed using ImProm Reverse Transcriptase (Promega), following the manufacturer's protocol, and miR-RT primer (Invitrogen). The miR-RT primer was: 5′-GCGAGCACAGAATTAATACGACTCACTATAGG(T)18VN-3′ . Real time PCR was performed using SYBR Green PCR Mix kit (Qiagen), according to the manufacturer's protocol [12], with the following primers: miR-130a, 5′-TTCACATTGTGCTACTGTCTGC-3′; miR-301a, 5′-GCTCTGACTTTATTGCACTACT-3′; miR-454, 5′-ACCCTATCAATATTGTCTCTGC-3′; universal primer, 5′-GCGAGCACAGAATTAATACGAC-3′; U6-F, 5′-CGCTTCGGCAGCACATATACTA-3′, U6-R, 5′-CGCTTCACGAATTTGCGTGTCA-3′. The relative level of expression of miR-130a/301a/454 was normalized to the internal control (U6) using the 2^−ΔΔCt^ cycle threshold method.

### Cell culture and transfection

The human colon cancer cell lines HCT116, HCT15, HT29, LoVo, and SW480 were obtained from the American Type Culture Collection (ATCC), and maintained in RPMI-1640 medium supplemented with 10% fetal bovine serum (FBS), penicillin and streptomycin, in a humidified atmosphere of 5% CO_2_ at 37°C. The miRNAs mimics and miRNA inhibitors were synthesized by GenePharma (Shanghai, China). MiRNAs transfections were performed using Lipofectamine 2000 (Invitrogen).

### Analysis of cell viability

The *in vitro* cell viability of HCT116 or SW480 cells was assessed using the Cell Counting Kit-8 (CCK-8, Dojindo, Japan) method. Briefly, spent medium was replaced with fresh medium containing 10 µl of CCK8 reagent at the indicated time periods posttransfection. The cells were then incubated at 37°C for 1 h and the number of viable cells was assessed by measurement of absorbance at 450 nm.

### Cell migration assay

HCT116 transfectants were serum-starved for 12 h in RPMI-1640 medium containing 0.1% FBS. Serum-starved cells were trypsinized and resuspended in RPMI-1640 containing 0.1% FBS, then 1×10^5^ cells were added to the upper chamber (8 µm pore size; Corning) of 24-well plates in serum-free medium (500 µl). After incubation for 24 h at 37°C in 5% CO_2_, the migrated cells on the lower surface of the membrane were stained with 0.1% violet staining solution for 30 min, and counted using an inverted microscope.

### Tumorigenicity assay in nude mice

All experiments involving animals were undertaken in accordance with the National Institute of Health Guide for the Care and Use of Laboratory Animals, with the approval of the Scientific Investigation Board of the General Hospital of PLA. The tumorigenicity assay was performed as reported previously [12]. Negative control or miR-130a/301a/454 mimic-transfected HCT116 or SW480 cells (1×10^7^) were suspended in 0.1 ml PBS, then injected subcutaneously into either side of the posterior flank of the same 4-week-old female BALB/c athymic nude mice. Eight nude mice were included in each group and tumor growth was measured daily using calipers. Tumor volume was calculated according to the following formula: volume = length×width^2^×0.5. The expression of miR-130a/301a/454 in tumor samples at the indicated times were detected using a qRT-PCR assay.

### 3′UTR luciferase reporter assay

The human Smad4 3′UTR luciferase reporter plasmid and plasmid containing the miR-130a/301a/454 target site deleted or mutated Smad4 3′UTR were constructed as described previously [13]. All constructs were confirmed by DNA sequencing. Luciferase reporter assays were performed, as reported previously [13]. Briefly, luciferase activities were measured at 48 h post-transfection using the Dual-Luciferase Reporter Assay System (Promega), following the manufacturer's instructions. Data were normalized by dividing *firefly* luciferase activity by *Renilla* luciferase activity.

### Immunoblotting

The lysed protein extracts were subjected to sodium dodecyl sulfate-polyacrylamide gel electrophoresis (SDS-PAGE), transferred onto nitrocellulose membranes, then blotted, as reported previously [24]. Anti-Smad4 antibody was purchased from Cell Signaling Technology. GAPDH antibody and the secondary antibodies were obtained from Santa Cruz Biotechnology.

### Statistical analysis

Data are shown as the mean ± s.d. Statistical comparisons between experimental groups were analyzed by Student *t*-tests, and a two-tailed p value<0.05 was considered to be significant. The correlation between miR-130a/301a/454 expression and the Smad4 protein level was analyzed using Pearson correlation coefficient analysis with r and p values, as indicated.

## Results

### MiR-130a/301a/454 is up-regulated in colon cancer tissues and cell lines

**Figure 1:**
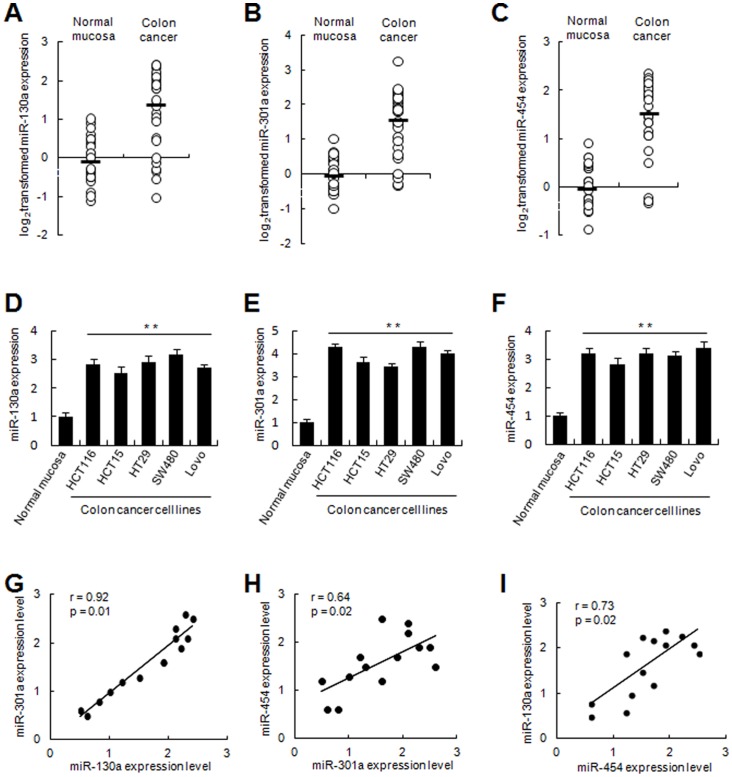
MiR-130a/301a/454 was up-regulated in human colon cancer tissues and cell lines. The expression of miR-130a/301a/454 was examined by qRT-PCR in 35 paired human colon cancer and adjacent normal mucosa tissues (A, B, C), and in colon cancer cell lines as indicated (D, E, F). The expression of miR-130a/301a/454 was normalized to U6 in each sample. Data are shown separately in human samples (A, B, C), or as the mean ± s.d. (n = 3) in cell lines (D, E, F). **, *p<0.01*. (G, H, I) Statistical analysis on the correlation between every two miRNAs among miR-130a/301a/454 was performed using Pearson correlation coefficient analysis. *r* and *p* values are shown as indicated.

To explore the roles of miR-130a/301a/454 in human colon cancer development, we detected the levels of expression in 35 pairs of human colon cancer and adjacent normal mucosa tissues. According to qRT-PCR analysis, the levels of miR-130a/301a/454 expression were significantly increased in tumor tissues compared to adjacent normal mucosa tissues (Fig. 1A–C). In addition, miR-130a/301a/454 was increased in colon cancer cell lines (HCT116, HCT15, HT29, SW480, and LoVo; Fig. 1D–F). Moreover, there was a positive correlation between every two miRNAs among miR-130a/301a/454 (Fig. 1G–I), showing a common expression profile in this miRNA family in colon cancer. These results suggest that miR-130a/301a/454 is up-regulated in colon cancer cells, which might be relevant to human colon cancer development.

### MiR-130a/301a/454 enhances cell viability and migration in colon cancer cell lines

**Figure 2:**
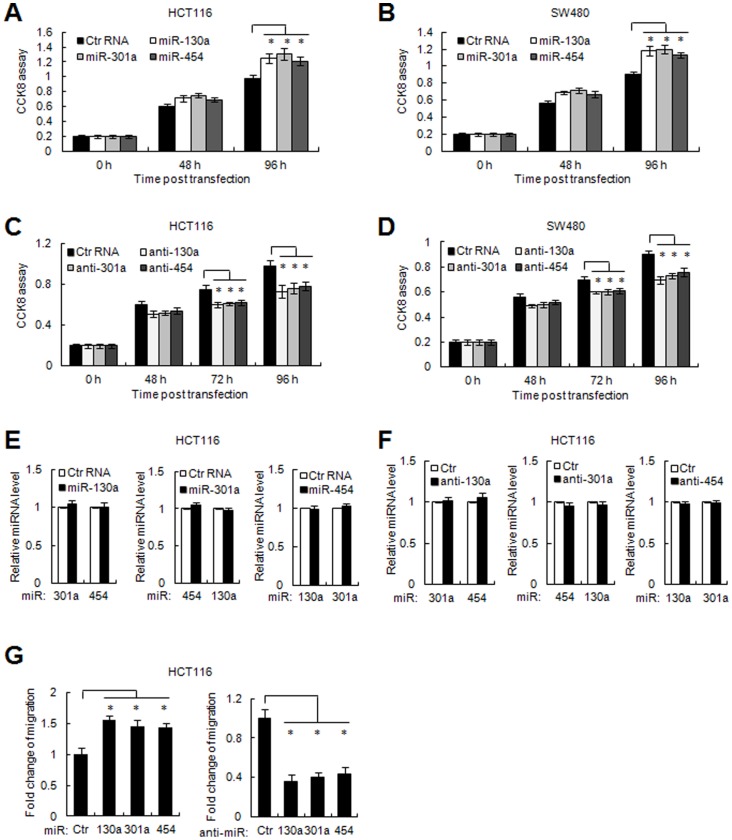
MiR-130a/301a/454 enhanced cell viability and migration. (A, B) Colon cancer HCT116 and SW480 cells were transfected with control RNA or miR-130a/301a/454 mimic as indicated. Cell viability was detected at the indicated time points post transfection using CCK8 assays. *, *p*<0.05. (C, D) The inhibitor of miR-130a/301a/454 was transfected in HCT116 and SW480 cells, and cell viability was detected at the indicated time points using CCK8 assays. *, *p*<0.05. (E, F) The levels of expression of every two miRNAs among miR-130a/301a/454 were detected by qRT-PCR assays when the mimic or inhibitor of the other miRNA was transfected in HCT116 cells. (G) HCT116 cells were transfected with control RNA, miR-130a/301a/454 mimic, or miR-130a/301a/454 inhibitor. The number of migrated cells was calculated and shown. Data are shown as the mean ± s.d. (n = 3) of one representative experiment. Similar results were obtained from three independent experiments. *, *p*<0.05.

The high expression of miR-130a/301a/454 in colon cancer tissues and cell lines prompted us to investigate the biological function of these miRNAs in colon cancer. The CCK8 assay showed that restoration of miR-130a/301a/454 significantly enhanced the proliferation of colon cancer HCT116 or SW480 cells (Fig. 2A and B), while over-expression of either inhibitor against miR-130a/301a/454 reduced cell viability in both colon cancer cells (Fig. 2C and D) These three miRNAs exhibited similar growth-promoting effects in colon cancers, and no mutual regulations among miR-130a/301a/454 were observed (Fig. 2E and F). Moreover, miR-130a/301a/454 promoted cell migration in HCT116 cells, as analyzed by the transwell cell migration assay (Fig. 2G, left panel). In contrast, knockdown of miR-130a/301a/454 suppressed cell motility in colon cancer cells (Fig. 2G, right panel). These observations suggest that miR-130a/301a/454 promotes colon cancer cell growth and migration, thus acting as oncogenic miRNAs in colon cancer.

### MiR-130a/301a/454 promotes tumorigenecity *in vivo*

**Figure 3:**
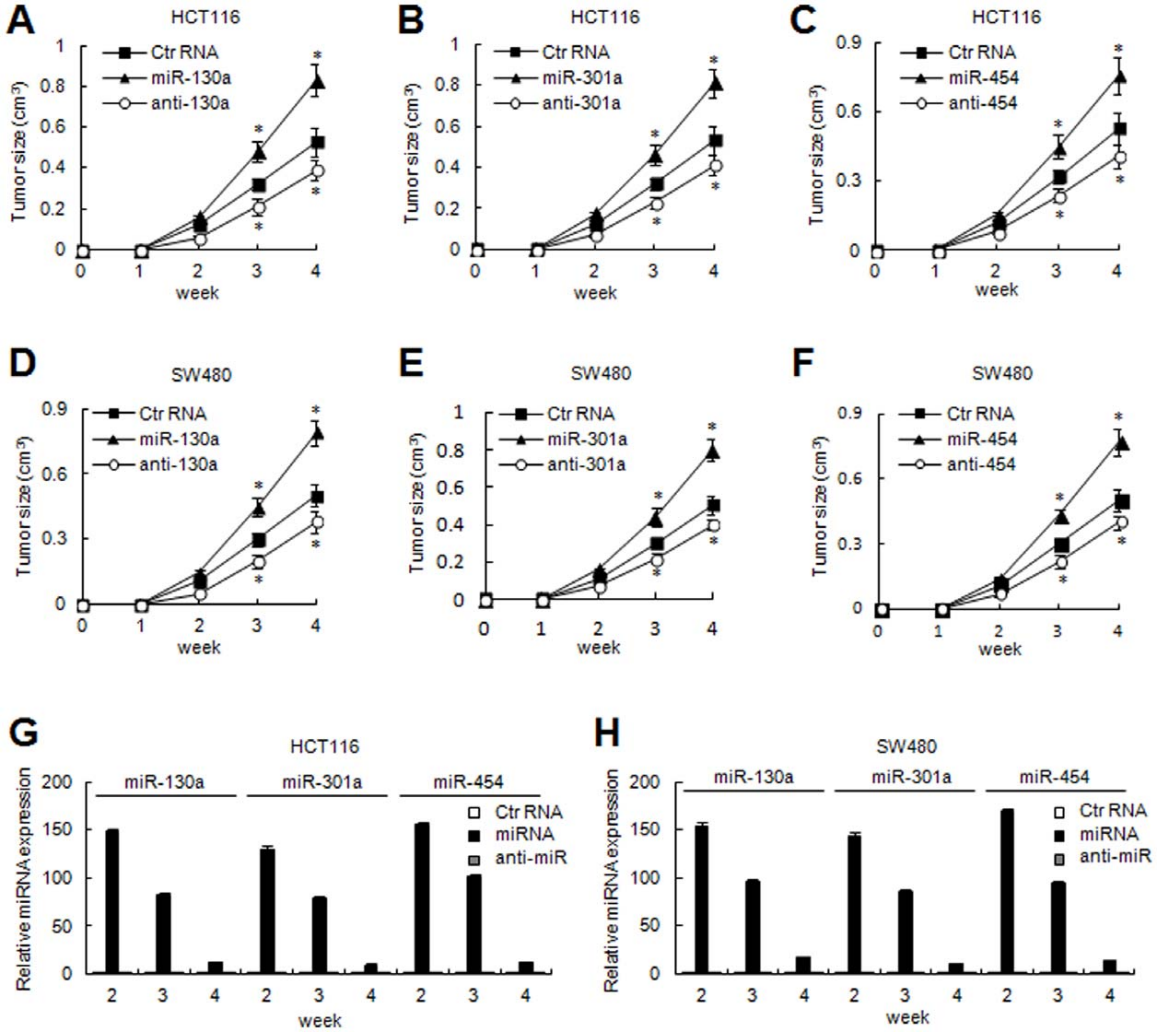
MiR-130a/301a/454 enhanced tumorigenicity. Effect of miR-130a/301a/454 on tumorigenicity in a nude mouse xenograft model. Control RNA, miR-130a/301a/454 mimic or miR-130a/301a/454 inhibitor-transfected colon cancer HCT116 cells (A, B, C) and SW480 cells (D, E, F) were injected subcutaneously into either side of the posterior flank of the same nude mice, respectively (n = 8 per group). The tumor growth curve is displayed as indicated. *, *p*<0.05. (G, H) Tumor tissues were collected from two mice in each group at the indicated times, and the levels of expression of miR-130a/301a/454 were examined by qRT-PCR assays.

We further confirmed the tumor-promoting effects of miR-130a/301a/454 *in vivo*. Negative control RNA, miR-130a/301a/454 mimic or inhibitor transfected colon cancer cells, were injected into eight nude mice, as described. As shown in Fig. 3, miR-130a/301a/454-transfected HCT116 and SW480 cells had rapid tumor formation compared to negative control transfectants, while knockdown of miR-130a/301a/454 caused delayed tumor formation (Fig. 3A–F). The levels of miR-130a/301a/454 expression were validated by qRT-PCR assays from the tumor samples at the indicated times (Fig. 3G and H). These results indicate that miR-130a/301a/454 significantly enhanced tumorigenicity of colon cancer cells in a nude mouse xenograft model.

### MiR-130a/301a/454 directly represses the expression of Smad4

**Figure 4:**
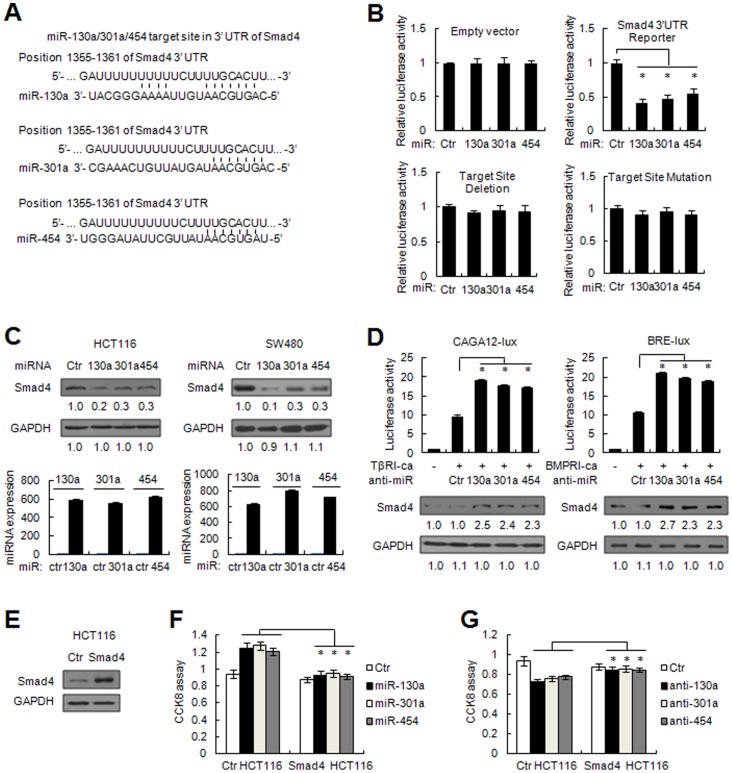
Tumor suppressor Smad4 is a direct target of miR-130a/301a/454. (A) Human Smad4 might be a molecular target of miR-130a/301a/454. The sequence alignments of miR-130a/301a/454 and its target sites in 3′UTR of Smad4, downloaded from TargetScan (http://www.targetscan.org) are shown. (B) HCT116 cells were co-transfected with human Smad4 3′UTR *firefly* luciferase reporter plasmid, miR-130a/301a/454 target sites deleted or mutated reporter construct, together with RL plasmids, control RNA, or miR-130a/301a/454 mimic, as indicated. After 48 h, *firefly* luciferase activity was measured and normalized by *Renilla* luciferase activity. (C) HCT116 and SW480 cells were transfected with control RNA or miR-130a/301a/454 mimic. After 48 h, human Smad4 and internal control GAPDH were detected by immunoblotting (upper panel), and the levels of miR-130a/301a/454 expression were measured by qRT-PCR assay (lower panel). The densitometry ratio for immunoblotting was shown. (D) HCT116 cells were co-transfected with the TGFβ reporter CAGA12-lux or the BMP reporter BRE-lux, together with RL plasmid, control RNA, or miR-130a/301a/454 inhibitor, and TβRI-ca or BMPRI-ca, as indicated. After 48 h, *firefly* luciferase activity was measured and normalized by *Renilla* luciferase activity. The endogenous expression of Smad4 and GAPDH were detected by immunoblotting. TβRI-ca, the constitutively active form of TGF-β receptor I; BMPRI-ca, the constitutively active form of BMP-receptor I. (E) Smad4 expression in empty pCDNA vector or Smad4-expressing plasmid stably-transfected HCT116 cells were detected by immunoblotting. (F, G) Control or Smad4 stably-transfected HCT116 cells were transfected with control RNA, miR-130a/301a/454 mimic (F), or miR-130a/301a/454 inhibitor (G), as indicated. Cell viability was detected 96 h post transfection using CCK8 assays. *, *p*<0.05.

On the basis of miRNA target prediction (http://www.targetscan.org), over a hundred genes are putative target genes for miR-130a/301a/454. As these miRNAs are able to enhance the tumorigenecity of colon cancer cells *in vitro* and *in vivo*, the target gene regulated by miR-130a/301a/454 can function as a tumor suppressor. Among the putative target genes, Smad4, an important tumor suppressor involved in TGF-β/BMP signaling, was shown to contain a conserved putative miR-130a/301a/454 target site based on TargetScan prediction (Fig. 4A). To determine whether or not Smad4 is directly targeted by miR-130a/301a/454 expression, we constructed luciferase reporter plasmids containing Smad4 3′-UTR or bearing deletion/mutation of the putative miR-130a/301a/454 target site. By co-transfection with miR-130a/301a/454 mimic, we showed that the luciferase activity of the wild type Smad4-3′UTR reporter was suppressed, while the target site deleted or mutated reporter failed to be targeted by miR-130a/301a/454 co-transfection (Fig. 4B). Of note, immunoblotting analysis showed that miR-130a/301a/454 markedly reduced the level of endogenous protein expression of Smad4 compared to negative control RNA in HCT116 and SW480 cells (Fig. 4C). Smad4 acts as a central transducer of transforming growth factor (TGF)-β and bone morphogenetic protein (BMP) signaling, because R-Smad forms a heteromeric complex with Smad4 and translocates into the nucleus to regulate gene transcription. We therefore sought to identify whether or not these miRNAs influence TGFβ and BMP signaling activities. miR-130a/301a/454 inhibitor was introduced in HCT116 cells transfected with the TGF-β reporter CAGA12-lux or the BMP reporter BRE-lux. As shown in Fig. 4D, miR-130a/301a/454 inhibitor markedly enhanced TGFβ and BMP responses when the constitutively active form of TGF-β/BMP-receptor I (TβRI-ca/BMPRI-ca) was co-expressed, accompanied by Smad4 up-regulation, suggesting that miR-130a/301a/454 can suppress TGF-β/BMP signaling by inhibiting Smad4 expression.

Furthermore, we determined whether or not the growth-promoting effects of miR-130a/301a/454 are mainly through targeting Smad4 expression. Smad4-expressing plasmid stably-transfected HCT116 cells were prepared, which transcribed Smad4 mRNA without the 3′UTR sequence. In these cells, Smad4 expression was confirmed to be increased compared to control cells (Fig. 4E); however, miR-130a/301a/454 no longer targeted Smad4 expression, as these cells transcribed Smad4 mRNA without the 3′UTR. Notably, the growth-increasing effects of miR-130a/301a/454 were significantly suppressed in Smad4-transfected HCT116 cells (Fig. 4F). Moreover, miR-130a/301a/454 inhibitor failed to suppress cell viability in these cells (Fig. 4G). These results further demonstrated that the growth-promoting roles of miR-130a/301a/454 were mainly through inhibition of Smad4 expression.

### Clinical correlation between miR-130a/301a/454 levels and Smad4 expression

**Figure 5:**
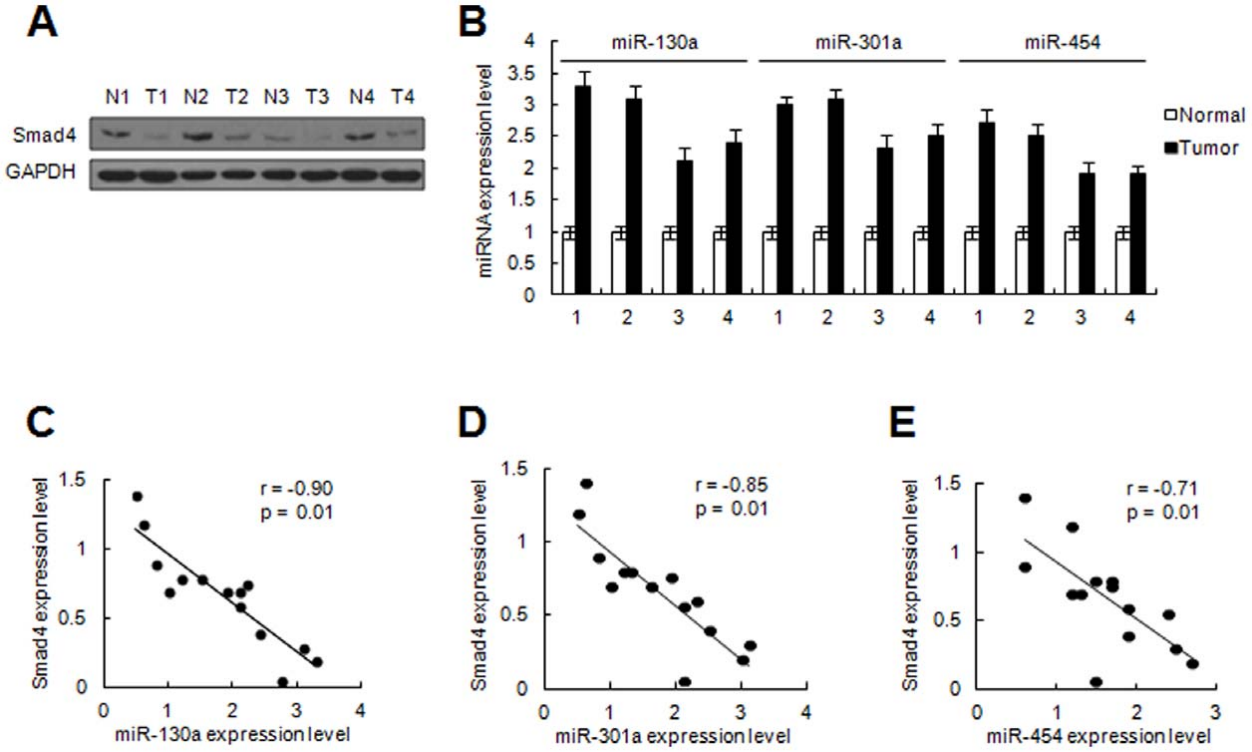
Inverse correlation between miR-130a/301a/454 and Smad4 protein level in colon cancer tissues. (A, B) Expression of Smad4 and GAPDH in samples of matched colon cancer and non-neoplastic mucosa tissues were detected by immunoblotting (A). miR-130a/301a/454 expression was examined by qRT-PCR (B). Four representative cases are shown. (C) The relative level of Smad4 or miR-130a/301a/454 expression was normalized to the internal GAPDH or U6 expression. Statistical analysis was performed using Pearson correlation coefficient analysis. Normalized miR-130a/301a/454 and Smad4 protein levels of expression are shown as standardized values. *r* and *p* values are shown as indicated.

To understand the clinical significance of miR-130a/301a/454 and its target in colon cancer, we determined the levels of miR-130a/301a/454 and protein expression of Smad4 in 14 pairs of matched colon cancer specimens by qRT-PCR and immunoblotting, respectively (Fig. 5A and B). As expected, Pearson correlation coefficient analysis suggested that Smad4 expression was inversely correlated with miR-130a/301a/454 expression in colon cancer tissues (Fig. 5C–E). These data support the notion that over-expression of miR-130a/301a/454 leads to Smad4 down-regulation, thus inhibiting TGF-β signaling-mediated cell growth suppression, which contributes to the development of colon cancer.

## Discussion

In the present study, we showed that miR-130a/301a/454 is up-regulated in colon cancer, and further demonstrated the proliferation-promoting effect of miR-130a/301a/454 in colon cancer cells. MicroRNA-130a/301a/454 is indicated as oncogenic miRNA in colon cancer development and progression, which is consistent with roles in other cancers, such as hepatocellular carcinoma, nonsmall cell lung cancer, chronic myeloid leukaemia, pancreatic cancer, and breast cancer [15]–[21]. However, miR-130a is down-regulated in chronic lymphocytic leukemia, and can modulate cell survival by inhibiting the autophagy program [22]. In addition, the plasma level of miR-301a is down-regulated in patients with sickle cell anemia, compared to normal controls, which is related to PAI-1 suppression [23]. The known patterns of expression, potential targets, and biological functions of this miR-130a/301a/454 family are summarized in Table 1 [15]–[23], [25]–[29]. The different expression features of miR-130a/301a may reflect the diverse roles of the miR-130/301 family in different types of cancer. Hence, deregulation of miR-130a/301a/454 exists in distinct types of cancer, and the roles of this miRNA family in carcinogenesis and progression cannot be simply concluded as a tumor suppressor or oncogene. Exploring the expression profiles and roles of miR-130a/301a/454 in colon cancer will greatly expand our comprehension for this important miRNA family on tumorigenesis.

**Table 1:** The known expression patterns, potential targets and biological functions of miR-130a/301a/454 family.

	Expression pattern	Cancer types	Targets	Function	References
miR-130a	upregulation	Nonsmall cell lung cancer	no functionally verified targets	associated with lymph node metastasis and poor prognosis	[15]
	upregulation	vascular endothelial cell	GAX, HOXA5	regulation of EC conversion to angiogenic phenotype	[16]
	upregulation	chronic myeloid leukaemia (CML)	CCN3	promote cell growth	[17]
	upregulation	chronic periodontitis	no functionally verified targets	related to inflammation	[26]
	downregulation	chronic lymphocytic leukemia (CLL)	ATG2B, DICER1	inhibit autophagy and trigger killing of CLL cells	[22]
miR-301a	upregulation	hepatocellular carcinoma (HCC)	GAX	increase proliferation, migration and invasion of HCC cells	[18]
	upregulation	pancreatic cancer (PC)	Bim	promote cell proliferation	[19]
	upregulation	breast cancer	FOXF2, BBC3, PTEN, and COL2A1	promote cell proliferation, migration and invasion	[20]
	upregulation	pancreatic cancer (PC)	NKRF	NK-kB activation	[21]
	upregulation	autoimmune demyelination	IL-6/23-STAT3	control autoimmune demyellination via regulating T-helper 17 immune response	[27]
	upregulation	chronic periodontitis	no functionally verified targets	related to inflammation	[26]
	upregulation	colonic cancer	no functionally verified targets	may participate in carcinogenesis	[28]
	downregulation	sickle cell anemia (SCA)	PAI-1	inhibit PAI-1 elevation	[23]
	no experimentally examined	brain	no functionally verified targets	brain developmental regulation	[29]
miR-301b	no experimentally examined	iPSC	GAX(MEOX2)	enhance iPSC generation	[25]
miR-454	no experimentally examined	no experimentally examined	no functionally verified targets	no experimentally examined	none

The TGF-β signaling pathway plays essential roles in numerous biological processes. Smad4, the pivotal transducer of the TGF-β and BMP signaling pathway, acts as an important tumor suppressor. Smad4 mutations or deletions have been widely observed in different types of cancer, such as colorectal, pancreatic, and breast cancers [30]–[32]; however, the detailed mechanism of Smad4 inactivation is limited. We demonstrated that miR-130a/301a/454 represses Smad4 expression through direct binding to 3′UTR, while we also noted that there are other miRNA consensus sites in the Smad4 3′-UTR (e.g., sites for miR-34a, miR-146a, and miR-199a, which have been identified as negative regulators of Smad4 in gastric cancer and glioblastoma) [33]–[35]. Therefore, we cannot rule out the possibility that these miRNAs also participate in the down-regulation of Smad4 expression in the development of colon cancer. Also, we cannot exclude the possibility that other potential targets of miR-130a/301a/454 may govern additional cancer pathways and promote the development of colon cancer, as a single miRNA is known to target multiple mRNAs. These hypotheses indicate the need for further studies to reveal the entire “targetome” of the miR-130/301/454 family in colon carcinogenesis and progression.

Induced pluripotent stem cells (iPSCs) can be generated by forced expression of transcription factors, including Oct4, Sox2, Klf4, and c-Myc [36]. Specific miRNAs have been shown to be involved in iPSC generation, such as miR-290 and miR-302 [37]–[39]. Recently, Pfaff et al. [25] showed that the miRNA family (miR-130/301/721) functions as an important regulator of iPSC induction by targeting the homeobox transcription factor, Meox2. The generation of iPSCs involves a process of mesenchymal-to-epithelial transition (MET), therefore factors inducing MET or blocking the epithelial-to-mesenchymal transition (EMT) by inhibiting TGF-β signaling play an essential role in cell reprogramming [40]. We have discovered a novel target of miR-130/301/454 family (Smad4), which has key function in TGF-β signaling, providing the possibility that the miR-130/301/454 family may control reprogramming by suppressing TGF-β/Smad4 activity. Thus, our studies have shed new light on the molecular mechanism and crosstalk of stem cell regulation and tumorigenesis.

In conclusion, our results confirm that the miR-130a/301a/454 family is a critical determinant in different kinds of cancer development and progression. Thus, the miR-130a/301a/454 family may represent a promising molecular target for the early detection of cancers.
